# Impact of Postgraduate Student Internships During the COVID-19 Pandemic in China

**DOI:** 10.3389/fpsyg.2021.790640

**Published:** 2022-01-07

**Authors:** Wenyi Zhang, Xiaozhong Lu, Derong Kang, Jiaxin Quan

**Affiliations:** ^1^School of Education, South China Normal University, Guangzhou, China; ^2^School of Business Administration, Guangdong University of Finance and Economics, Guangzhou, China; ^3^Institute for Advanced Study of Education Development in Guangdong-Hong Kong-Macao Greater Bay Area, South China Normal University, Guangzhou, China; ^4^School of Psychology, South China Normal University, Guangzhou, China

**Keywords:** COVID-19, postgraduate student, internship, anxiety, depression

## Abstract

To understand the impact of the COVID-19 pandemic on postgraduate students’ internships in China, 911 students from different regions of China were surveyed through online questionnaires. Among the postgraduate students surveyed, 48.51% of which believed that the pandemic had its greatest impact on colleagues interaction, and 59.60% believed that the pandemic had a strong impact on practical skills. In total, 31.8% of postgraduate internship programs were impacted by COVID-19. The proportions of respondents having severe, moderate, and mild anxiety levels were 1.42%, 4.72%, and 15.92%, respectively; and the rates of severe, moderate, and mild depression were 1.64%, 10.86%, and 21.84%, respectively. ANOVA found that major, degree type, and degree of impact of the pandemic on colleague interactions and improved practical abilities all affected postgraduate mental health. The findings suggest that the mental health of postgraduate students should be monitored during a pandemic, and targeted psychological counseling should be offered. Postgraduate internships should be emphasized as to ensure a smooth internship process during a pandemic period. Psychological counseling and assistance should be provided to those whose internships were affected by the pandemic, and programs should be set up to aid postgraduate students in adapting to the new internship and employment conditions brought on by the “new normal” of pandemic prevention and control.

## Introduction

With the spread of COVID-19, a large part of the global economy is in a state of “shutdown.” The UN Department of Economic and Social Affairs stated that “Against the backdrop of COVID-19, the world economy is expected to shrink by 3.2% in 2020, marking the strongest contraction since the Great Depression of 1930.” China’s economy has also received serious shocks, with many micro-, small- and medium-sized enterprises facing the risk of layoffs and closures. The severe economic situation has had the most direct impact on the postgraduate student population through changes in employment pressure. The number of workforce employed in the first half of 2020 was significantly lower than that in previous years. According to China’s Ministry of Education (2020) National Education Development Statistics Bulletin, 728,600 postgraduate students graduated in 2020, showing an increase of 13.89% compared with 2019 (China’s Ministry of Education, 2020). Moreover, owing to the worldwide spread of the pandemic, postgraduate students originally scheduled to study abroad had to postpone or even cancel their plans, and some of them rejoined the domestic job market. The sudden increase in employment pressures has affected the psychological expectations of school students, and this change has made the next stage of employment competition even more intense.

Since the second half of 2020, the direct impact of COVID-19 on postgraduate employment has gradually decreased as the effectiveness of epidemic prevention and control started to emerge, and China’s economy started to gradually recover. According to 2021 Graduate Employment Trend Report published by Boss Zhipin^1^, a popular Chinese job recruitment website, the demand for spring recruits in 2021 increased by over 40% year-on-year. However, in the current job market, internship experiences are essential for postgraduate students to secure a job. Postgraduate internships generally are placed on long holidays such as summer and winter vacations. However, during the winter and summer vacations, population density tends to rise, resulting in the increase of the risk of sporadic COVID-19 outbreaks. In fact, in 2019, after the virus was first detected in Wuhan, sporadic outbreaks occurred throughout China during the following winter and summer holidays. There is an inevitable contradiction between the needs of postgraduate students for internships and the need to strengthen pandemic control during long holidays. In the context of the normalization of pandemic control, COVID-19 continues to adversely affect postgraduate employment. To reduce the risk of COVID-19 spreading, companies and organizations are trying to adopt new forms of online work and work from home options. This change has also been applied to postgraduate internships. Thus, online internship has become an option that postgraduate students have to consider.

According to Lazarus and Folkman’s psychological stress theory (Folkman and Lazarus, 1988), when encountering a great crisis, individuals will have a series of physiological, behavioral, and emotional stress reactions that are based on their own cognitive assessment. In the face of public health crisis, the public is the first to experience emotional fluctuations and negative feelings, such as anxiety, anger, helplessness, and panic. Stress reaction under crisis conditions can trigger defensive behaviors and such behaviors have adaptive significances. However, if negative emotions cannot be regulated and relieved, then the individual is exposed to a longer state of stress, which can damage the physical and mental health of the individual, easily leading to depression, anxiety, and other psychological problems.

Anxiety and depression are common psychological problems and conditions for the public during the pandemic (Ren et al., 2020). For postgraduate students, the initial COVID-19 outbreak was at the end of the year, a critical time for postgraduate internships and employment. Over the next 2 years, prevention and control of the pandemic will impact the mental health status of postgraduate students in a manner that is different from other student groups. According to statistics, the total number of students enrolled in higher education in China is 41.83 million, among which postgraduate students accounting for approximately 7.5%, including 0.46 million Doctoral degree students and 2.67 million Master degree students. Compared with other groups of students, the postgraduate group has the following characteristics: (1) Postgraduate students are mainly adults between the age 20–30; (2) They have received higher education; (3) They have a clearer perception about realistic pressure (Moss et al., 2021); (4) Their body and mindset are in upward stages of development; (5) They usually face higher expectations from employers, especially on the quality and length of their internship experiences (Barry et al., 2018); and (6) They have both greater autonomy and stronger pressure on internship selection other students.

In summary, research on internships and mental health of the postgraduate students during the pandemic is urgent and necessary. Many scholars have conducted research on the mental health of students in the context of the pandemic. Research by Wei et al. (2020) demonstrated that children and adolescents in severely affetcted areas, or those whose family members are infected with COVID-19, will experience negative emotions, such as anxiety and depression, and severely affected individuals may be overly scared, restless, and confused. The impact of the pandemic has been evident, and a higher detection rate of negative emotions in college students, such as depression and anxiety, has been reported compared with before the pandemic (Cao et al., 2020; Chang et al., 2020; Chen et al., 2020; Khan et al., 2020; Li et al., 2020; Og et al., 2020; Son et al., 2020; Niu et al., 2021).

However, there is a lack of research into the mental health of postgraduate students as a specific group. Some scholars have also conducted research on the internship practices of Chinese college students during the pandemic. Shen et al. (2020) explored the “cloud practice” teaching strategy for traditional Chinese medicine subjects during the pandemic; Chai et al. (2021) brought forward suggestions for online internship-related teaching. Even though the above studies provided us with insights into internship placement and practical training of university students during the COVID-19 pandemic, they have the following limitations: (1) Most studies focused on students of specific majors, such as medicine or management, therefore, the issues discussed and conclusions drawn were limited to specific groups of students; (2) Most surveys targeted undergraduates or senior students, and did not take into consideration the differences between the differences in internship placements between postgraduate and undergraduate students; and (3) the research samples have geographical limitations; consequently, it was not possible to represent the large population of colleges and universities in mainland China.

Therefore, while investigating the levels of anxiety and depression among Chinese postgraduate students during the pandemic, current study also investigated the extent of the impact specifically on the internship activities. The aim was to understand the internship activities and mental health status of postgraduate students in the context of both the COVID-19 pandemic and the “new normal” of pandemic prevention and control measures, to provide an important reference for targeted psychological counseling, and also to provide advice on postgraduate internship and employment guidance.

In addition to being the first country to detect the coronavirus and achieve effective control of the virus, China is also the first major economy to recover. As postgraduate students are representatives of highly qualified job-ready groups, it is necessary to look into problems associated with postgraduate student internship and their mental health status. It will provide an important reference for economic recovery and job promotion in other major economies of the world.

## Materials and Methods

### Participants

A total of 911 postgraduate students (96.20% valid response rate) were randomly selected from 33 provinces or autonomous regions in China *via* the popular professional survey website WenJuanXing^2^. All respondents participated voluntarily and signed informed consent. This study was approved by the local Ethics Committee of the School of Psychology, South China Normal University (SCNU-PSY-2021-021). The sample included 195 male students (21.40%) and 716 female students (78.59%); 834 master-degree students (90.18%) and 77 doctoral-degree students (8.45%); and 279 academic degree postgraduates (30.62%) and 632 professional degree postgraduates (69.37%).

### Data Analysis

In this study, we used R 4.0.2 for data analysis. *T*-test and one-way analysis of variance (ANOVA) were used to explore the main factors influencing depression, anxiety, and social anxiety score.

### Measures

#### Self-Rating Anxiety Scale

The Self-Rating Anxiety Scale (SAS) was used to assess individual’s anxiety in the recent week (Zung, 1971). The scale included 20 self-rating items out of which 5 items were scored in the reverse order. The scale used 4-point Likert scale with scores on responses ranging from 1 (occasionally) to 4 (always). The sum of the item scores was referred to as the total rough score, then it was multiplied by 1.25 to obtain the standard score. The scale had a Cronbach’s alpha equal to 0.85. A standardized scoring algorithm was used to define anxiety symptoms: scores 50∼59 indicated mild anxiety; scores 60∼69 indicated moderate anxiety; and scores > 69 indicated severe anxiety.

#### Self Rating Depression Scale

The Self Rating Depression Scale (SDS) was used to assess individual’s depression in the recent week (Zung, 1965). The scale included 20 self-rating items out of which five items were scored in the reverse order. The participants responded on the 4-item Likert scale with scores ranging from 1 (occasionally) to 4 (always). The total rough score was the sum of scores of all items, and the total rough score was multiplied by 1.25 to obtain the standard score. The Chinese version of the scale had a Cronbach’s alpha equal to 0.88. A standardized scoring algorithm was used to define depression symptoms: scores 53∼61, 62∼71, and above 72 indicated mild, moderate, and severe depression, respectively (Wang et al., 1986).

#### Questionnaire on the Impact of COVID-19 on Postgraduate Students Internship

Questionnaire on the impact of covid-19 on postgraduate student’s internship was designed to measure how postgraduates’ internship were affected by the pandemic. Respondents reported the level of impact on their internship caused by the pandemic on a 4-point Likert scale with severity-centric options, namely “no impact,” “little impact,” “moderate impact,” and “strong impact.”

The three scales used in this study generally maintain their original versions. The Harman one-way test The test for common method bias using the Harman one-way test means showed that the first factor has had an interpretation rate of variance of 39.4%, not below 50%, so there which indicated there is was no common method bias.

### Ethical Considerations

The ethic committee of South China Normal University approved the study. All participants voluntarily gave their informed consent to participate in the study after being informed about the purpose of the study. The procedures of this study complied with the provisions of the Declaration of Helsinki regarding research on human participants.

## Results

### Anxiety and Depression

The descriptive results of SAS and SDS are shown in the table below. Table 1 shows that the postgraduate anxiety scores and depression scores during the pandemic were higher than their corresponding Chinese norms, and the differences were highly statistically significant (*p* < 0.001).

**TABLE 1:** Postgraduate anxiety and depression description statistics.

	Participants		Norms		*t*
	*M*	SD	*M*	SD	
SAS	42.80	10.03	29.78	5.46	72.91***
SDS	47.67	11.21	41.88	10.57	28.97***

***p < 0.001.

Specifically, according to the Chinese Norms, 201 (22.06%) and 313 (34.35%) postgraduate students were identified with symptoms of anxiety and depression, Incidence rates of mild, moderate, and severe anxiety were 15.92, 4.72, and 1.42%, respectively. and those of mild, moderate, and severe depression were 21.84, 10.86, and 1.64%, respectively.

Prior to the outbreak, some researchers had studied the utility of SAS and SDS for postgraduate students: Wu et al. (2013) used SAS and SDS to evaluate anxiety and depression in 177 graduate students and found that the corresponding rates were 10.7 and 24.3%, respectively. Wang (2017) conducted a survey on 941 students pursuing a professional degree and found that the rates of detection of anxiety-related and depression-related symptoms were 2.6 and 8.5%, respectively, both of which are lower than the rates observed in the current study. With regard to other studies on the mental health status of Chinese college students during the COVID-19 pandemic, Chang et al. (2020) conducted a survey on 3,881 Cantonese college students and found that the incidence of anxiety among university students was 26.60%; Mao et al. (2020) found depression- and anxiety-related symptoms in medical-major postgraduate students were 38.75% and 24.17%, respectively. Other researchers detected a higher rate of anxiety and depression in post-graduate students during the pandemic than before the pandemic (see Table 2).

**TABLE 2:** Comparison of rates of anxiety and depression in other studies and this study.

References	*N*	Research Objects	Rate of anxiety	Rate of depression
Wu et al., 2013	177	Medical college graduate students	10.7%	24.3%
Wang, 2017	941	Professional degree graduate students from 3 universities in Chongqing	2.6%	8.5%
Chang et al., 2020	3,881	Cantonese college Students	26.60%	–
Mao et al., 2020	240	Medical graduate students	24.17%	38.75%
Current study	3,137	Chinese postgraduate students with internship experience	22.06%	34.35%

### Mental Health Status of Postgraduate Students Based on Demographics Various Categories

Demographic variable such as gender, degree type, and major were used as independent variables; anxiety and depression scores were used as dependent variables.

Statistical analysis of the differences in demographic variables were performed using independent *t*-test for binary factors (i.e., gender and degree type) or ANOVAs for multi-level factors (i.e., major).

#### Gender

The *t*-tests were conducted on anxiety and depression scores of postgraduate students of different genders. There were no significant differences in anxiety scores and depression scores among postgraduate students of different genders (Table 3). Jiang et al. (2016) conducted a meta-analysis on the mental health of postgraduate students in China, and had concluded that gender has no significant influence on postgraduate student mental health. The findings of current study, consistent with most previous studies, indicate that the mental health status of both males and females were affected by the pandemic.

**TABLE 3:** The difference in demographic variables of all study variables (M ± SD).

Demographic variable		*n*	Statistics	Anxiety	Depression
Gender	Male	195		42.09 ± 10.15	46.92 ± 11.43
	Female	716		42.75 ± 9.77	47.76 ± 10.91
			*T*	0.04	0.08
Degree type	Academic degree	276		43.08 ± 10.32	47.97 ± 11.24
	Professional degree	632		42.20 ± 9.42	47.25 ± 10.83
			*t*	7.86**	1.71
Professional Type	Science and engineering	109		42.92 ± 9.51	47.99 ± 11.26
	Liberal arts	159		43.42 ± 10.25	48.98 ± 11.53
	Social science	643		42.36 ± 9.83	47.19 ± 10.84
			*F*	0.86	3.40*

*p < 0.05 and **p < 0.01.

#### Academic Degree and Profession Degree

The *t*-tests were conducted for postgraduate students of different degree types. As shown in Table 3, significant differences occurred in anxiety scores (*t* = 7.86, *P* = 0.005 < 0.05). Academic degree postgraduate students had significantly higher anxiety scores than professional degree postgraduate students, but the degree type did not significantly influence depression scores (*t* = 3.39, *P* = 0.066 > 0.05). Academic degree postgraduate students may be affected more by the pandemic because of their different academic systems, stricter graduation requirements and the need for more academic support, resulting in relatively higher anxiety level, but not depression level.

#### Major

ANOVA was used to examine the differences between the scale scores of postgraduate students from different majors (Table 3). There were significant differences in depression scores (*F* = 3.40, *P* = 0.034 < 0.05), and the highest depression scores were among postgraduate students majoring in liberal arts (48.98 ± 11.53), which were far higher than Chinese norm (41.88 ± 10.57). However, there were no significant differences in anxiety scores among postgraduate students from different major, indicating that major did not significantly affect anxiety.

### Relationship Between the Degree Affected on Internships and Mental Health

This study surveyed 911 postgraduate students with internship experience. Since these students already had previous internship experience, they should be able to recognize the potential impacts on internships brought by the pandemic. Thus, this study aims to investigate how changes in internship plans influence students’ anxiety and depression levels. The following table shows the anxiety and depression scores related to changes in the internship program.

As shown in Table 4, during the pandemic, 209 postgraduate students delayed their internship programs, and 81 students canceled. And 31.80% of the postgraduate internship programs were affected by the pandemic and did not proceed as planned. A total of 478 postgraduate internships, or 52.46% of the total, were back on a regular schedule. On the one hand, these data showed that the pandemic in China has been effectively controlled and that the resumption of work and production is proceeding in an orderly manner. On the other hand, nearly one-third of postgraduate students still failed to complete their internships, which also revealed that postgraduate internship programs had been greatly impacted by the pandemic. Postgraduate students whose internships were canceled had significantly higher anxiety scores (44.94 ± 12.34) and depression scores (50.43 ± 12.54) than other postgraduate students. The mental health status of students whose internship was on track delayed or those who did not plan on participating in internships exhibited no deviation from the sample mean. This indicates that postgraduate students whose internship programs were canceled have significant mental health risks that deserve the attention of mentors, parents, and counselors.

**TABLE 4:** Changes in different internship programs and comparison of scale scores (M ± SD).

Changes in Internship plans	*n*	Anxiety	Depression
On track	478	42.73 ± 10.05	47.40 ± 11.30
Delayed	209	42.70 ± 9.11	48.28 ± 10.51
Cancel	81	44.94 ± 12.34	50.43 ± 12.54
No plans	143	41.94 ± 9.78	46.16 ± 10.94

The anxiety and depression scores of 478 postgraduate students whose internships were normally conducted during the pandemic are shown in Table 5.

**TABLE 5:** Comparison of different internship sites and scale scores (*M* ± *SD*).

Internship site	*n*	Anxiety	Depression
Company	440	42.58 ± 10.02	47.48 ± 11.31
Home	38	43.50 ± 9.62	49.92 ± 10.63

As shown in Table 5, 440 postgraduates’ internships were taken place at a company. This further illustrates the gradual normalization of life in China and the smooth resumption of work and production. Only 7.95% of the 478 regular postgraduate students completed their internships at home during the pandemic, indicating that online internships are still not accepted by the majority of postgraduate students and enterprises.

Postgraduate students who did internships at home had higher levels of anxiety and depression than postgraduate students who intern in a company, indicating that postgraduate students working from home experienced more serious mental health problems. This may be related to the lack of recognition for online internships, with postgraduate students being skeptical about whether these internships provide the work experience and professional skills on a par with traditional internships. Additionally, during online internships, postgraduate students are unable to communicate face-to-face with superiors, colleagues, and clients, making it difficult to get direct social interactions and positive feedback (Thoits, 2011).

In this survey, “Interaction with colleagues” and “Practical ability improvement” were addressed, and specific items were set up to inquire about the impact of the pandemic on the internships of postgraduate students. The different degrees of impact and corresponding mental health status are shown in Table 6.

**TABLE 6:** Comparisons of the scores of postgraduate students that were affected to different degrees.

Item		*n*	Statistic	Anxiety	Depression
The impact on interactions with colleagues	No impact	206		41.33 ± 8.88	45.43 ± 10.78
	Little impact	272		41.48 ± 9.28	46.71 ± 10.54
	Moderate impact	369		43.50 ± 9.81	48.93 ± 11.00
	Strong impact	73		48.41 ± 14.07	51.44 ± 14.03
			*F*	11.63***	7.84***
The impact on the improvement of practical skills	No impact	165		40.89 ± 8.87	45.26 ± 11.04
	Little impact	203		41.60 ± 9.04	45.49 ± 10.51
	Moderate impact	386		43.74 ± 10.00	49.22 ± 10.64
	Strong impact	157		44.05 ± 11.94	49.27 ± 12.73
			*F*	4.97**	8.84***

**p < 0.01, and ***p < 0.001.

As shown in Table 6, 369 and 73 out of 911 postgraduate students considered that the pandemic had a “moderate impact” and “strong impact” on their interactions with colleagues. In addition, 386 and 157 students considered that the pandemic had a “moderate impact” and “strong impact” on the improvement of practical skills. In summary, more than half of postgraduate students believe that internship activities have been significantly affected by the pandemic.

ANOVA was conducted with the degrees of influence as the independent variables, and anxiety and depression scores were used as the dependent variables. There were significant differences in the degree of impact of COVID-19 on colleague interactions and on anxiety and depression scores (Table 6). Using the LSD method to conducted multiple comparisons, postgraduate students whose degrees of impact were “moderate impact” and “strong impact” had higher anxiety and depression scores. This suggested that the greater the impacts on the interactions with colleagues and the improvement of practical skills, the more serious the psychological problems in postgraduate students.

## Discussion

This study shows that 20.91 and 33.88% of postgraduate students amid COVID-19 had anxiety and depression symptoms, respectively, and them had significantly higher anxiety and depression scores than Chinese norms. Postgraduate students’ mental health problems are worsening compared with pre-pandemic data. During the pandemic, people’s concerns and fears over their own situations became more prominent, and the strict prevention and control measures across the country created a tense environment. Additionally, the impacts of the pandemic hampered the economy and industry, as well as postgraduate studies, research, internship, and employment opportunities (Moreira de et al., 2018). Such stresses also cause psychological problems, such as anxiety and depression (Shi et al., 2016).

A comparative analysis of the mental health of postgraduate students in different majors and degree types revealed that mental health problems are more serious among academic degree postgraduate students and students majoring in liberal arts. These research results are consistent with previous study by Wang et al. (2020). Academic degree postgraduate students have greater academic pressure, higher dissertation standards, and less vocational skill training than professional degree postgraduate students. The pandemic disrupted their original study plans and intensified job competition, resulting in greater anxiety (Moore and Lucas, 2020).

Therefore, during the pandemic, universities should consider the practical difficulties faced by academic degree postgraduate students, adjust course arrangements appropriately, maintain open and direct communication channels, and provide guidance to postgraduate students (Wang, 2015; Santamaría et al., 2021). In addition, we should also pay attention to employment guidance for academic degree postgraduate students, and cultivate their scientific research and practical abilities. Only in this way can students build up more confidence in the face of drastic changes under pandemic conditions.

Students majoring in liberal arts showed more severe depression, possibly because these students are more empathetic, more emotionally sensitive, and are more susceptible to social circumstances. Therefore, universities and supervisors should pay attention to the mental health of liberal arts postgraduate students, devise specific programs to ensure clear and open communication and to provide psychological counseling and guidance in a targeted manner.

This study found that postgraduate students whose internships were canceled due to the pandemic had severe anxiety, depression and psychological health risks. With China’s current postgraduate training scheme and employment situation, individuals whose internship program was canceled does not only face reduced employment competitiveness, but also the risk of not fulfilling graduation requirements. Successful internships in the future will depend on COVID-19 prevention and control measures, and the urgency of time will undoubtedly increase the stress for senior students as their graduation season is approaching (Ryan et al., 2021). Therefore, in view of the fact that a large number of postgraduate internship programs cannot be carried out smoothly due to the pandemic, universities and employers should explore new ways to cooperate, provide more flexible internship methods, and help postgraduate students to complete successfully in the period of normalization of pandemic prevention and control through online internship and remote internship plan.

In the face of the COVID-19 public health crisis, attention should be paid to the mental health problems of postgraduate students and various measures should be taken to intervene actively and effectively. Universities, mentors, counselors, and other relevant personnel should: (1) Actively guide postgraduate students to understand their mental health, encourage postgraduate students to seek help in case of psychological problems, and avoid other negative effects, such as post-traumatic stress disorder; (2) Establish monitoring and early warning system for psychological problems, and improve online and offline psychological counseling service system; and (3) Devise specific and targeted mental health programs according to the different characteristics and needs of various postgraduate students groups.

Faced with postgraduate internship problems during a pandemic, universities should establish new mechanism of cooperation and communication with employers. Both sides should work to provide various options, such as field internships, online internships, remote training, or building internship practice platform. In addition, postgraduate students should also consciously communicate with colleagues and participate in practical training and work practice.

Colleges and universities should pay attention to the mental health of postgraduate students to prevent their emotional state from worsening because of employment pressure. Additionally, universities should guide them to take positive views of problems. With the easing of the pandemic, there will be more internship opportunities in the near term. At present, a comprehensive set of skills and, good job plans should be developed. Only in this way can students better meet the challenges of future job competition.

## Limitation

There are still a limitation for our study that should be noted. Given the fact that the pandemic had spread worldwide, it is extremely difficult or even impossible to find Chinese postgraduate students who did not experience such interruptions for comparison. As a cross-sectional survey, there was no follow-up period for the participants, which means we didn’t yet know enough about the changes of mental health of postgraduate students during the pandemic.

## Data Availability Statement

The raw data supporting the conclusions of this article will be made available by the authors, without undue reservation.

## Ethics Statement

The studies involving human participants were reviewed and approved by the Ethic Committee of South China Normal University. The patients/participants provided their written informed consent to participate in this study.

## Author Contributions

XL, DK, and JQ selected the topic and made some modifications in the manuscript. WZ collected the data and wrote the manuscript. All authors contributed to the article and approved the submitted version.

## Conflict of Interest

The authors declare that the research was conducted in the absence of any commercial or financial relationships that could be construed as a potential conflict of interest.

## Publisher’s Note

All claims expressed in this article are solely those of the authors and do not necessarily represent those of their affiliated organizations, or those of the publisher, the editors and the reviewers. Any product that may be evaluated in this article, or claim that may be made by its manufacturer, is not guaranteed or endorsed by the publisher.

## Footnotes

1
www.zhipin.com

2
www.wjx.cn
